# Real-time dose reconstruction and dose coverage forecasting using the magnetic resonance linear accelerator^☆^

**DOI:** 10.1016/j.phro.2026.100910

**Published:** 2026-01-26

**Authors:** Peter R.S. Stijnman, Pim T.S. Borman, Stijn Oolbekkink, Cornel Zachiu, Martin F. Fast, Bas W. Raaymakers

**Affiliations:** Department of Radiotherapy, University Medical Center Utrecht, the Netherlands

**Keywords:** Dose evaluation, Real-time, Image-guided radiotherapy

## Abstract

**Background and purpose:**

Intrafraction patient motion during radiotherapy treatment affects the delivered dose, thereby, deviating from the planned dose. To monitor the deviation, we propose a workflow capable of performing and displaying real-time dose calculations during treatment with a magnetic resonance linear accelerator (MR-linac). Additionally, the workflow can forecast the accumulated dose and enables evaluation of dose guidance in real time for triggering automatic plan adaptation or intrafraction drift corrections (IDCs).

**Materials and methods:**

Information on the treated anatomy, rigid translations or three-dimensional (3D) volumes, and the linac state were collected to perform clinical grade dose calculations. Real-time dose reconstructions and the accumulated dose forecasts were performed for two dynamic scenarios. The first, rigid translations derived from two-dimensional orthogonal MR images where the calculated dose was compared to diode measurements. The second, deformable dose accumulation using deformable image registration, based on 3D MR imaging and compared to a dose film measurement.

**Results:**

The real-time dose reconstruction had a maximum standard deviation error of 2.3 % with the measured dose, compared to 13.1 % for the planned dose. The workflow maintained real-time performance using a single graphics processing unit up to 92 % of the time that the beam was on. Finally, dose coverage forecasts were updated every 6.5 s at the start of treatment, with the update frequency increasing towards the end of treatment.

**Conclusion:**

We demonstrated the feasibility of displaying real-time delivered dose distributions and forecasted dose coverage using an MR-Linac. This enables triggering automatic plan adaptations or IDCs.

## Highlights

• Real-time dose reconstruction during magnetic resonance-linear accelerator treatment.
• The latency of the presented workflow was 150 ms.
• The phantom measurements were within a 2.3  % standard deviation of calculation.
• The presented workflow updated accumulated dose and target coverage every 6.5 s.
• The presented workflow supported rigid and deformable dose accumulation.

## Graphical abstract

**Figure:**
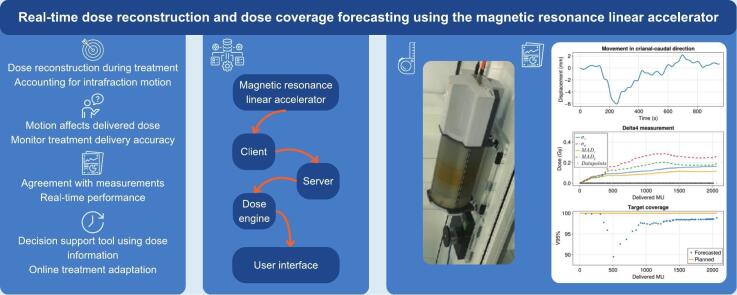

## 1 Introduction

Since the introduction of image-guided radiotherapy, information on patient anatomy has been used to improve the accuracy of treatment delivery. Well known methods include; position verification [1], [2], offline or online treatment plan adaptation [3], [4], [5], [6], beam gating [7], [8], [9], and multileaf collimator (MLC) tracking [10], [11], [12]. These approaches have improved treatment efficacy by increasing target coverage or organ of interest (OoI) sparing. However, the anatomical information used in these approaches is a surrogate for the actual delivered dose, which is the main quantity of interest to precisely know how efficient the treatment delivery has been [13].

For position verification, a single snapshot of the patient anatomy is used for a geometric correction minimizing the effect of the interfraction motion on the delivered dose, but discarding the impact of the intrafraction motion. The same holds for online treatment plan adaptation on the anatomy of the day. When the patient anatomy in previous fractions does not correlate well with the planning imaging, offline treatment plan adaptation can be considered. Here, new images are acquired and a new treatment plan is generated to minimize the effect of interfraction motion. Furthermore, both beam gating and MLC tracking only consider the intrafraction motion as a geometric change, without considering the impact on the delivered dose.

In this study, we developed a workflow that leverages two- or three-dimensional (2D, 3D) images acquired with a magnetic resonance linear accelerator (MR-Linac) to calculate the delivered dose in real time and forecast the dose coverage at the end of the fraction. The focus of this work is that performing these calculations can be done within the timeframe of the treatment. In the future, this real-time dose information could be utilized as part of a decision support tool to trigger intrafraction drift correction (IDC) based on target coverage, or to enable real-time plan adaptation.

## 2 Materials & methods

### 2.1 Workflow infrastructure

**Fig. 1:**
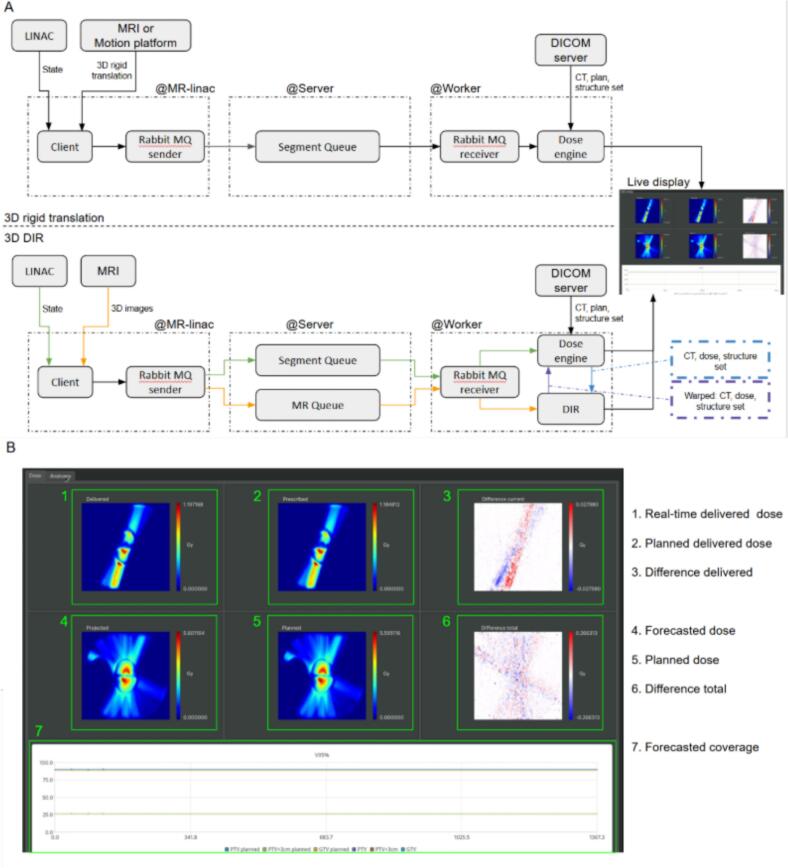
A) An overview of the workflow for the 3D rigid translations and the 3D deformable image registration (DIR). At the linac a computer received the status of the linac and either the motion trace or images from the MR device. Here the linac state and the motion information are synced. This data is packaged and submitted to a queue on a server running an AMQP (advanced message queueing protocol, more details in the supplementary material), for the DIR a separate queue for the MR images is used. A worker retrieves the data and starts a dose engine to calculate the delivered dose and reports the information on a live display, seen in more detail in B).

An overview of the workflow is shown in Fig. 1. The workflow consists of three parts, namely the MR-linac, a server, and a remote worker. Details on the server can be found in the supplementary material. The other two parts are described in more detail below.

At the MR-linac, a computer was connected to both the network of the MR device and the linac. From the linac, messages containing the state of the linac were obtained at a 5 Hz rate. These messages contained the jaw and MLC leaf positions, the gantry angle, and the amount of delivered monitor units (MU). We interpreted the information contained in a message as a control point. Between two control points if the beam was on, a segment was created for which the delivered dose was calculated.

From the MR device, we acquired either 3D translations from the comprehensive motion management (CMM, from Elekta AB, Sweden) or 3D volumes of the treatment site [14]. The 3D translations were obtained at a 5 Hz rate by tracking the target inside two orthogonal 2D balanced gradient echo T1-weighted MR images at a 3.7 × 3.7 × 5 mm^3^ resolution. These translations were synchronized with the linac state and used to rigidly translate the patient anatomy for the dose calculation.

Alternatively, 3D MR images of the treated anatomy were used to warp the reference data. The registration and warping was performed by an in–house developed deformable image registration (DIR) algorithm, Evolution, that can register multi-modal images [15]. Additional information on the DIR algorithm used can be found in the supplementary material. These 3D MR images or the rigid translations and the delivered plan segments were sent from the control room to the server.

The final part of the workflow was the remote worker that received the segments and the MR images. For dose calculations a clinical-grade dose engine (GPUMCD, Elekta AB, Sweden) was used [16], [17]. The dose was calculated on a 3 mm isotropic grid with a statistical uncertainty of 5 % per calculation on a graphics processing unit (GPU), the NVIDIA RTX A5000.

The planning CT and structure set that contained delineations of the patient were loaded into the dose engine and adapted to the current patient anatomy using either the rigid translations or the DIR.

The workflow calculated three dose distributions:

1. The real-time dose reconstruction;
2. The planned dose up to the same MU (i.e. ignoring the intrafraction motion);
3. The dose that will be delivered on the latest static anatomy calculated using the remainder of the treatment plan (i.e. the forecast).

The first two dose distributions were used to obtain a difference between the delivered and planned dose, revealing the impact of the intrafraction motion on the dose. Finally, the first and third dose distribution were added to obtain a forecast of the final dose distribution on the latest anatomy for assessment of deviations in dose coverage.

### 2.2 Measurements

**Fig. 2:**
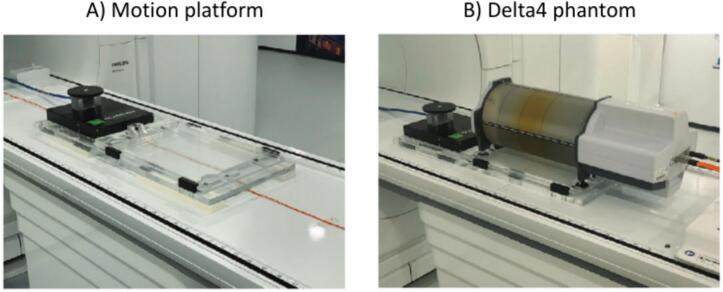
Measurement setup for the 3D rigid translation workflow test. Picture A) shows the motion platform that was used to introduce the three different motion traces. Picture B) shows the Delta4 phantom placed on top of the motion platform.

**Fig. 3:**
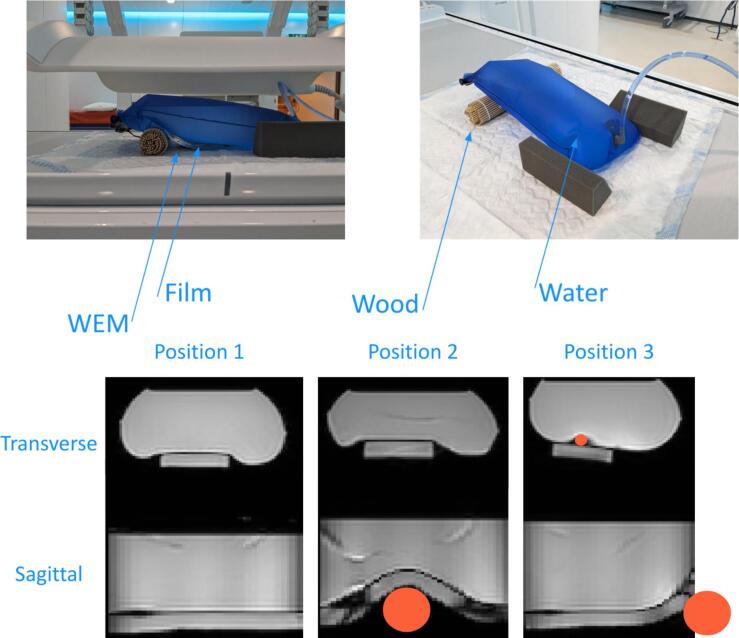
Measurement setup for the deformable workflow test. A bag was filled with water and placed on the table. Under the bag is a piece of water equivalent material (WEM) with dose film in between. Fish oil tablets (i.e. small orange circle in the transverse slice of position 3) were placed next to the ends of the dose film to find the correct position of the film inside the magnetic resonance images. Treatment delivery was paused twice to place a wooden roll (i.e. larger orange circles in the sagittal slices) under the setup to displace the water and deform the film, resulting in a different measured dose distribution compared to the planned dose. (For interpretation of the references to colour in this figure legend, the reader is referred to the web version of this article.)

We used two different measurement setups. The first, Fig. 2, was to demonstrate the workflow using 3D rigid translations, which could be integrated more easily into the current clinical workflow using the CMM. The second, Fig. 3, was to demonstrate the workflow to perform deformable dose accumulation using 3D MR images.

For the rigid measurement, we used a Delta4 phantom + MR (ScaniDos AB, Sweden), which contained diodes capable of time-resolved dose readouts at 40 Hz [18]. This phantom was placed on top of a Quasar Motion MR Platform (IBA Quasar, Canada) to introduce rigid translations [19]. Both were placed inside a 1.5 T Unity MR-linac (Elekta AB, Sweden). For this measurement we used the self-reported motion from the motion platform as input for the 3D rigid translation because this phantom cannot be scanned using the MR device. In a separate measurement with a QUASAR MRI^4D^ motion phantom (Modus Medical Devices inc., London, Ontario, Canada) the workflow with a 3D rigid motion trace obtained from the CMM workflow was tested.

Measurements were performed using 3 different motion traces supplied to the motion platform while delivering a prostate IMRT plan. The first was a static scenario to test the workflow and make sure that the dose reconstruction, planned dose, and measured dose corresponded with each other. The second motion trace was a linear drift from 0 to 1 cm in the cranial-caudal direction. Finally, the third motion trace was derived from a prostate cancer patient treated with a 5 fraction 7.25 Gy SBRT plan.

For the comparison, we focused on the diodes that received between 60 % and 85 % of the maximum dose. These diodes were located in the high dose gradient and therefore displayed the largest dose change as a result of the motion introduced. To show the impact of the rigid motion, we plotted the standard deviation and the mean absolute difference of the difference between the measured and the planned dose (σ_P_, MAD_p_) and between the measured and calculated delivered dose (σ_c_, MAD_c_). Finally, for the forecasted dose distribution we compared the planned with the forecasted dose coverage (i.e. V95 %) of a clinical target volume (CTV) that was inside the high dose region.

The setup for the deformable measurement was less conventional, a plastic 3 l hydration bag used for long-distance running or hiking, was filled with water. Underneath the bag, we placed a slab of water equivalent material (WEM) (Medisynt, Biberstein, Switzerland), and in between a 5 × 10 cm^2^ sheet of EBT3 GafChromic film (Ashland, New Jersey, USA). Furthermore, two fish oil tablets, 0.8 mm diameter by 2.5 mm height and containing 1000 mg of fish oil, were placed at the ends of the film for localization. The MR images were acquired with a 3D balanced turbo field echo cine sequence with a 2 × 2 × 2.2 mm^3^ resolution. The TR and TE were 3.7 ms and 1.86 ms, respectively. Furthermore, a compressed sensing reduction factor of 1.4 was used to achieve a dynamic scan time of 9.9 s.

To introduce deformations, we placed a wooden roll underneath the hydration bag. During delivery of a 10 × 10 cm^2^ beam to the phantom, treatment delivery was paused at two time points to position the wooden roll at different locations. The dose accumulation was performed by registering these states with DIR.

Dose comparisons were made between the calculated accumulated dose, the planned dose, and the measured dose at the film location. Gamma pass rates were calculated using a global normalization and a minimum dose threshold of 5 % of the maximum planned dose. The films were scanned approximately 24 h after irradiation, and the optical density was converted to relative dose maps using an in-house developed software tool written in MATLAB [20], [21].

During the measurements we generated a log file containing the timings of the workflow. The time required to reconstruct the dose and to perform the DIR were logged to demonstrate the ability of the workflow to keep real-time performance under varying linac duty cycles. The linac duty cycle was defined as the percentage of the beam on time divided by the time between the first beam on and the last beam off. Moreover, the time required to forecast the dose was logged and used to plot the frequency of the target coverage updates.

## 3 Results

**Fig. 4:**
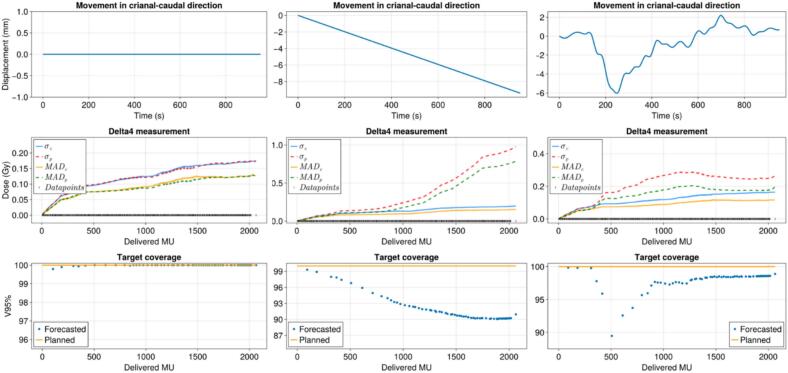
This figure displays the results of the rigid workflow. The columns of figures are the three separate measurements that were performed. The top row indicates the different motion traces that were used: static, linear drift, and patient–derived drift. The second row shows the standard deviation of the difference and the mean absolute difference between the planned and measured dose (red and green) and the live calculated and measured dose (blue and yellow). These values are calculated up until the delivered MU they are plotted against. Furthermore, the datapoints are shown by black vertical lines in the bottom. The bottom row displays the V95 % of the CTV that was drawn inside the high-dose region. The V95% was calculated for the planned dose (yellow line) and each of the forecasted dose volumes during the treatment (blue dots). (For interpretation of the references to colour in this figure legend, the reader is referred to the web version of this article.)

The results for the rigid workflow test are shown in Fig. 4. Without motion present, σ_P_ and σ_C_ overlapped with a maximum value of 0.17 Gy (i.e. 2.3 % of the fraction dose of 7.5 Gy). Furthermore, the forecasted coverage remained equal to the planned coverage, with a maximum deviation of 0.2 %.

For the linear drift, we observed that σ_P_ increased throughout the treatment with a final value of 0.98 Gy or 13.1%. While σ_c_ remained similar to the static scenario with a maximum of 0.19 Gy or 2.5 %. The forecasted coverage decreased to 90.1 % target coverage.

Finally, for the patient-specific derived prostate drift motion, initially, σ_P_ increased to 0.29 Gy or 3.9 %. Around halfway through the treatment σ_P_ decreased again. The maximum value for σ_c_ was 0.16 Gy or 2.1 %. The target coverage initially dropped to 89.5 % and increased during the treatment back to 97.5 %. An overview of the uncertainty budget can be seen in the supplementary material.

**Fig. 5:**
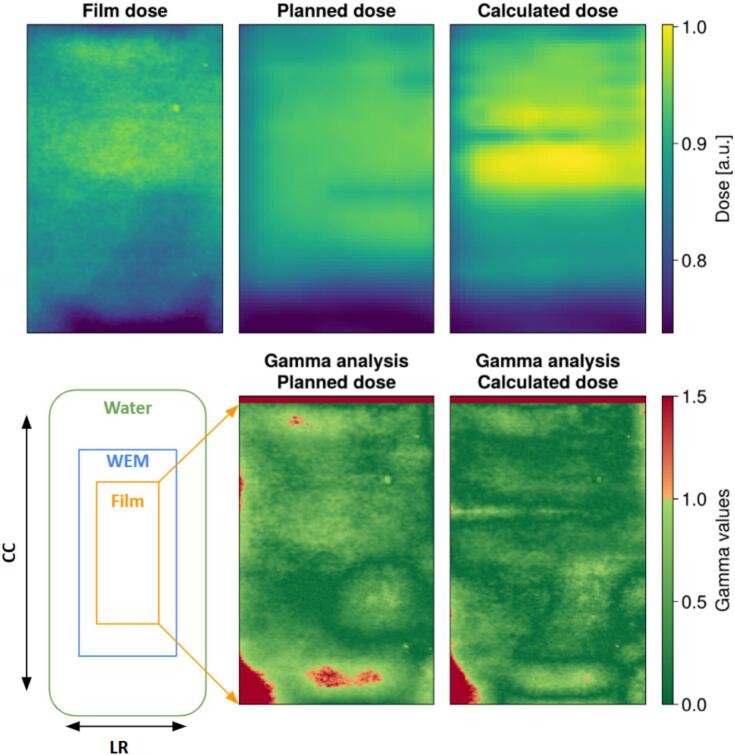
This figure shows the results of the deformable workflow. The top left shows the dose that was measured on the dose film. Next to that is the planned dose distribution at the location of the film, and the top right image shows the live calculation, using the deformable dose accumulation, for this same location. On the bottom row are the 2 %/2 mm gamma analysis for the dose distribution directly above them.

The results for the deformable workflow test are shown in Fig. 5. The 2 % /2 mm gamma pass rate increased from 95.5 % for the planned dose to 98.2 % for the calculated dose.

**Fig. 6:**
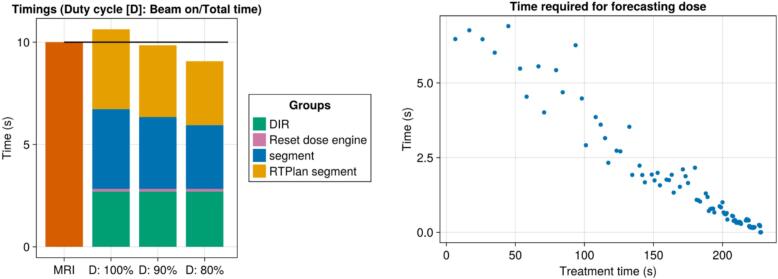
Timings for a computer with a single GPU utilized during the measurements. The left image shows the timings for the separate calculations of the workflow within the 10-second timeframe of the MR-acquisition for different duty cycles of the linac. At a 100 % duty cycle, the workflow will trail the live treatment for the deformable workflow. For a duty cycle of 92 % or lower the workflow can maintain real-time performance. For the rigid workflow, the “DIR” and “Reset dose engine” times are 0 s, therefore, always keeping up with the live treatment. The right figure shows the intervals for the forecasted dose. The rate at which these are calculated increases as the treatment is delivered because less of the treatment needs to be forecasted.

The timings for the workflow are shown in Fig. 6. Currently, the workflow could maintain real-time performance up to a 92 % duty cycle. On average calculating a dose segment required 82 ms and the forecast took approximately 6.5 s to calculate. However, this time decreased as the treatment was delivered since fewer segments remained.

Finally, latency between receiving the linac state and displaying the dose in the user interface was observed to be less than half a second. Exactly timing this was rather complex since there were multiple computers, all on different networks/time servers, involved. For the deformable workflow, the DIR halted the dose calculations for about 3 s, after which the workflow quickly caught up to the live scenario.

## 4 Discussion

In this work, we developed a workflow to perform real–time dose reconstruction and forecast the dose on an MR-linac. For this, we can either use rigid translations obtained from tracking a target on 2D images, or we can perform DIR using 3D images of the patient’s anatomy for the dose accumulation.

This real-time dose reconstruction could be used for multiple purposes. First, the difference between the forecasted dose and the planned total dose could be used to estimate the precision of the treatment during the treatment itself or trigger an IDC based on dose rather than geometric guidance. By incorporating the forecasted target coverage, clinicians could make more informed decisions that directly relate to treatment precision. Second, the difference in the calculated delivered dose and the planned dose up until that point could be used for real-time treatment adaptation.

The moment to trigger an IDC depends on the clinic and their goals for treatment accuracy and how often they want to perform a treatment adaptation. For our offline treatment checks we investigate patients that have a single fraction with a CTV V95 % coverage under 95 %. For triggers on OoIs we would suggest starting with the planning guidance and defining a threshold of 5–10 % and monitor those values.

The challenge of implementing a trigger based on the real-time and forecasted dose is highlighted in Fig. 4, bottom right. Retrospectively an IDC is not necessarily warranted since the coverage at the end is 97.5 %. While during the treatment at the 400–500 delivered MU point, with a projected target coverage well below 95 %, a treatment plan adaptation would be triggered.

There have been other studies that showed the possibility of performing real-time dose calculations. One such example was the work by E. Persson et al that showed it was possible to perform dose calculations using GPUMCD to obtain a real-time dose distribution [22]. The main differences with this work is that we integrated the workflow with the MR-linac system, performed dosimetry measurements, and added DIR to accumulate the dose. Another work was that of E. Hewson et al that showed real-time dose calculations to optimise the MLC aperture shape to correct for intrafraction motion [23]. This was tested *in silico* and used line-of-sight calculations. Finally, the works from S. Skouboe [24] and K. Klucznik [25], showed real-time dose reconstructions including rigid motion obtained from CBCT imaging of fiducial markers. However, they used a pencil beam model to calculate the dose. Therefore, the work presented here expanded upon the previous work by including a clinical grade dose engine and DIR.

In the presented workflow, the planned dose could be computed beforehand for treatment plans where only the amount of delivered MU changes within a segment. For treatment plans where other values change within a segment (e.g. VMAT) this would not work and the correct start and end point for these changing values are required. To keep the workflow more generally applicable we chose not to compute the planned dose beforehand.

From Fig. 4, we observed that the motion had an effect on the dose distribution and that when we accounted for this motion the calculation corresponds better with the measured dose. However, there was still a small deviation between the real-time dose calculations and the measured dose. This is in part a result of the Monte Carlo dose engine that was used, and in part from the uncertainties in the measurement setup. The accuracy of the Monte Carlo dose calculation could be improved by reducing the variance. At a 5 Hz rate, the delivered MU at a maximum dose rate of 420 MU/min is 1.4 MU. Significantly lower than the average treatment plan dose segment. When three or more of these live dose segments were within one treatment plan segment, the resulting noise in the calculated dose distribution compared to a treatment plan calculated with a statistical uncertainty of 3 % will be lower (i.e., 5 %/√3 < 3 %).

In the proposed workflow it is easy to add computation power, the exact details on this are described in the supplementary material. These additional resources can be used to either further improve the statistical uncertainty of the Monte Carlo simulation by calculating more particle interactions. Furthermore, the additional computing power could also be used to increase the update frequency of the forecasted dose, or to keep real-time performance for linac treatments that have a duty cycle higher than 92 %, i.e. VMAT treatment plans [26].

Given the maximum dose rate of 420 MU/min for the MR–linac we can express the latency of our workflow as the maximum amount of MU we could possibly trail the live treatment by. The 82 ms required for a dose segment translates to 0.57 MU. At the start of the treatment it took at most 6.9 s to forecast the dose, trailing live treatment by 48.3 MU. When we performed the DIR workflow the dose calculations were halted during the DIR step for 3 s, thereby trailing the live treatment by 21 MU.

For the deformable measurement the location we placed the wooden roll underneath the water bag, thereby displacing the water and thus shortening the radiologic length and increasing the dose to which the film was exposed at those locations. This behaviour was better captured with our proposed workflow as is indicative of the dose maps in Fig. 5 and the increased gamma pass rates.

A limiting factor with this phantom is that it is simplistic compared to the clinical setting. Furthermore, the film was placed at an interface, that of water and the WEM, which was the focus of the DIR algorithm used. In future work, we wish to test the deformable measurement with a standardized phantom and setup and integrate some form of DIR quality assurance. This would enable us to perform the absolute dosimetry for a more precise indication of the deformable dose accumulation performance. Another limiting factor of the deformable workflow is the rate at which we obtained the MR images, every 10 s. This is too slow to track breathing and cardiac motion. A possible solution would be to substitute the 3D volumes for 2D orthogonal slices and have a model predict the 3D deformation vector fields from the 2D images [27] or reconstruct the 3D motion directly from acquired k-space data using MR-MOTUS [28]. Additionally, the time it takes to perform the DIR would need to be reduced.

In conclusion, we demonstrated a workflow that can calculate the real-time delivered dose for an MR-linac. Furthermore, we forecasted the total dose and the dose coverage given anatomical updates obtained from rigid translations or 3D MR images. This could in the future be used for triggering automatic plan adaptations or intrafraction drift corrections.

## CRediT authorship contribution statement

**Peter R.S. Stijnman:** Conceptualization, Methodology, Software, Validation, Formal analysis, Investigation, Writing – original draft, Writing – review & editing, Visualization. **Pim T.S. Borman:** Conceptualization, Methodology, Software, Investigation, Writing – review & editing. **Stijn Oolbekkink:** Conceptualization, Methodology, Validation, Formal analysis, Writing – review & editing. **Cornel Zachiu:** Software, Writing – review & editing. **Martin F. Fast:** Conceptualization, Writing – review & editing, Funding acquisition. **Bas W. Raaymakers:** Conceptualization, Writing – review & editing, Supervision, Funding acquisition.

## Declaration of competing interest

The authors declare the following financial interests/personal relationships which may be considered as potential competing interests: Our department has a research agreement with Elekta AB on the development of adaptive strategies for the CBCT-linac. PS is working on a grant for this purpose, which is partly financed by Elekta AB. Elekta AB had no role in the preparation, review, or approval of the manuscript or the decision to submit the manuscript.

UMC Utrecht acknowledges funding by the Dutch Research Council (NWO) through project no. 20827 (ART-PIVOT).
