# Supplementary material for: Real-time dose reconstruction and dose coverage forecasting using the magnetic resonance linear accelerator
Source: Phys Imaging Radiat Oncol. 2026 Jan 26;37:100910. doi: 10.1016/j.phro.2026.100910 (PMC12906094; doi:10.1016/j.phro.2026.100910)
Supplement: Supplementary Data 1 [file mmc1.pdf]

Supplementary information for "Real-time dose reconstruction and dose coverage forecasting using the magnetic resonance linear accelerator" by Stijnman P et al.

Deformable image registration details

The deformable image registration (DIR) algorithm that we used was EVolution. This is a non-rigid multi-modal registration method that aims to maximize edge alignment between the set of images it registers. This algorithm has been tested for MR to MR (i.e. also with different contrasts), CT to MR, CT to CT and CBCT to CT image registration [1,2].

The mean target registration error is in the order of the voxel size of the registered scans. For example the mean registration error for a 1.25 by 1.25 by 4 mm^3 MR to 0.65 by 0.65 by 4 mm^3 CT was 3.3 mm with a standard deviation of 1.84 mm [1].

To obtain the results in the manuscript we used the following settings

Table S1: DIR settings.

Registration dimensions	128 x 128 x 128 voxels
α	0.35
Maximum number inner iterations	1000
Maximum number outer iterations	10
Cutoff cost function	0.01

Server settings

On a computer that is both connected to the linac control room network and the main hospital network we installed a RabbitMQ server (version 4.0) [3]. RabbitMQ is an implementation of the advanced message queuing protocol (AMQP). The concept is that the server can host different "exchanges" with "queues" connected to them. Then a "publisher" can send messages to an exchange with a "routing key" and a "subscriber" can listen to one of the queues. A simple overview can be seen in Figure S1. The exchanges and the queues live on the server, and the publisher(s) and subscriber(s) have to connect to the server to start sending or receiving data.

In our case the publisher is a computer that is connected to the network of the MRI and the linac. It receives the state of the linac and creates a segment of two consecutive states where there has been MU delivered. The relevant information gets written to a message to be sent to the exchange. This is done using ProtoBuf (proto2), which takes the data of the message and serializes it to a string [4]. Then we add a routing key in the form of "segment.*name_of_linac*" and publish it on the exchange. The exchange inspects the routing key and sees that it is dealing with a segment for which we want to calculate the dose, and it

sees the name of the linac from which this segment came. Using this information the exchange selects the correct Queue and forwards the message.
For the MR images a similar setup is made. The images get serialized to a string per slice using ProtoBuf. The routing key now has the form of “image.*name_of_linac*”. The data is again published to the exchange which selects the correct queue based on this routing key. The subscriber obtains the new MR images. In Figure S2 our configuration for the RabbitMQ server is shown.

In Figure S2 we show the setup for the three MRI-linacs at our department. This illustrates the ease of use for this configuration, where an additional treatment machine can simply be added by adding a queue to the exchange and attaching the correct routing keys. The same holds for adding additional workers (i.e. computational power).

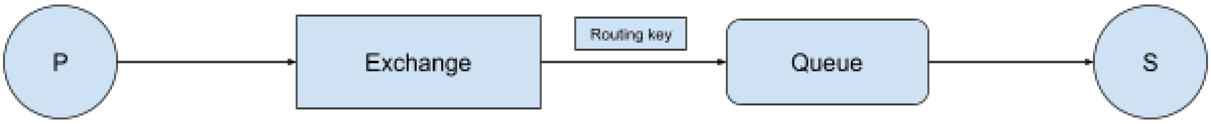

Figure S1. A simple diagram on how an AMQP server could be configured. Here “P” is a publisher that sends messages (i.e. data) with a routing key to an exchange. The exchange inspects the routing key and forwards the message to the correct queue. The subscriber “S” looks at the queue in intervals to receive any data that is currently queued.

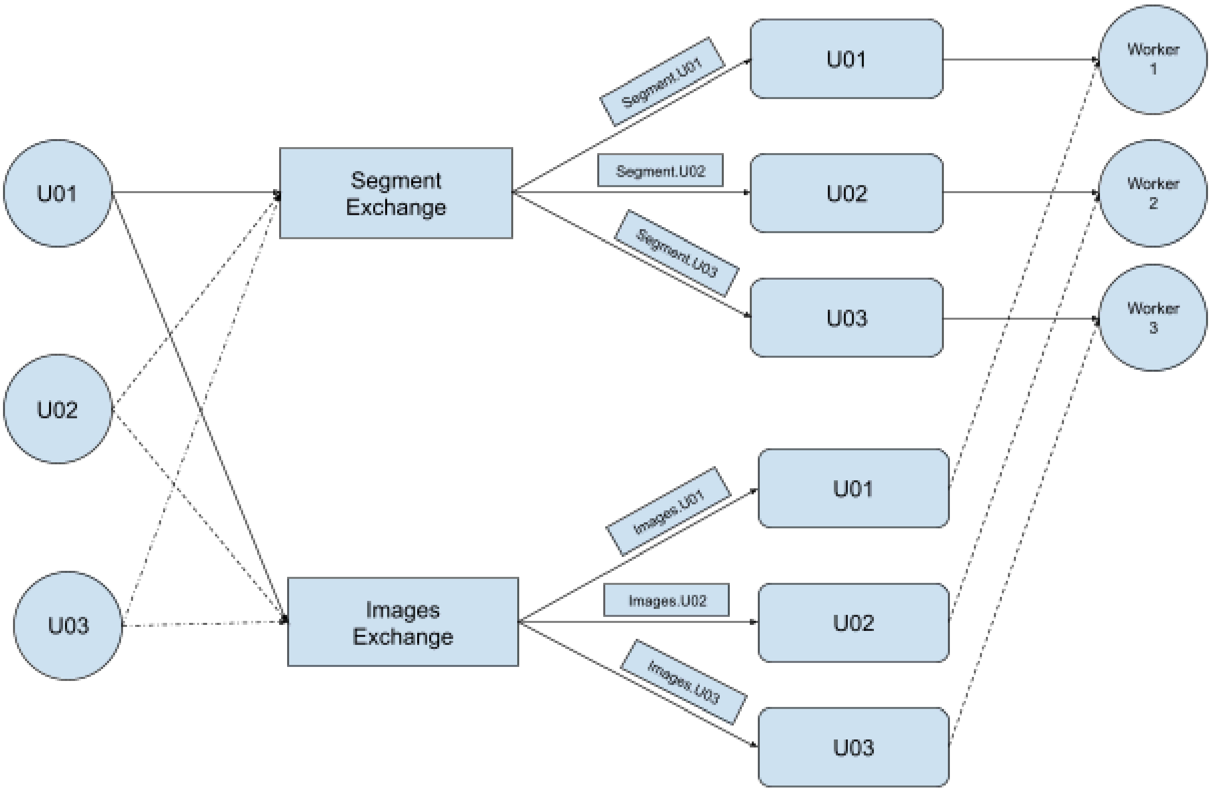

Figure S2. A more detailed overview of the used RabbitMQ server. Here the exchanges and Queues live on the server, while the publishers “U01/2/3” are the computers in the control room that collect the linac and MRI data. The workers are the computers fitted with a GPU to perform dose calculations using GPUMCD, DIR using EVolution, and display the results to the user.

Workflow uncertainty budget

For the rigid results the standard deviation for our workflow was within 2-3% below are some sources of and their contribution to the buildup in this error.

The Monte Carlo variance was set to 5% per segment reported by the linac (5 Hz rate).
The position was reported every 100 ms. For the linear drift case this equates to a maximum deviation of the actual and reported position of 9mm / 950 s treatment time * 0.1 s maximum lag = 0.00095 mm = 0.95 μm.

The diode calibration has a smaller than 1% measurement uncertainty after 10 time samples [6]. This means that for segments that take longer than 250 ms the uncertainty of the diode measurement is smaller than 1%.

From the same paper we can also observe that for a TRF/log file calculation the uncertainty in the dose calculation becomes smaller than 1% after 30 samples. These were calculated using an 8% statistical uncertainty compared to the 5% used here. This would entail that 12 samples would result in a dose uncertainty of smaller than 1%, this equals 480 ms.

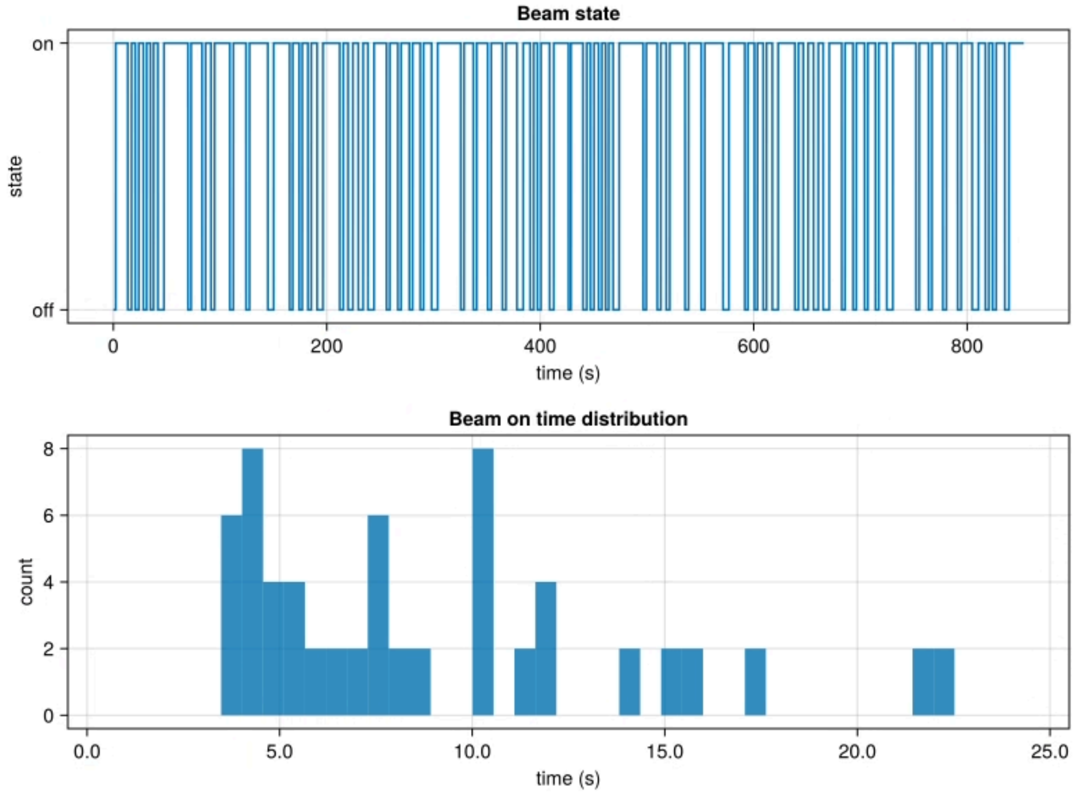

Figure S3: The beam on time obtained from the treatment record file (TRF) which records the state of the linac every 40 ms. The top plot shows when the beam is on during the

treatment. The bottom histogram shows the length of the segments during the measurement. With the shortest segment having a beam on time of 3.84 seconds.

From Figure S3 we can observe that for this specific plan the smallest segment measured in beam on time was 3.84 seconds. Which is well above the 480 ms required for a smaller than 1 % measurement uncertainty.

The DIR accuracy is not part of this measurement, since it was used for the dosimetric film measurement. As mentioned in the manuscript, the DIR QA is still an active research area and something we wish to integrate in the future.

Given the values above, we believe that a 2-3% agreement is justified. Similar to how clinically 2%/2mm or 3%/3mm gamma pass rates are used.

Workflow pseudocode

In this section some pseudocode is given to indicate when particular calculations are performed, and what data is used to perform those calculations. Overall dose calculations are started once the segments are received from the server. Forecasts are made continuously if there is not already a forecast calculation running. Starting with the client at the MR-linac:

Client @MR-linac: main thread

```
function main(file)
{
   // parse relevant network settings
   settings = parse_input(file);

   // establish connection as publisher to server
   connect_to_rabbitMQ();

   // spawn threads to obtain linac and MR data
   linac_thread(connect_to_linac, settings.linac_address);
   if (settings.using_DIR)
      mr_thread(connect_to_mr, settings.mr_address);
   else // CMM or phantom
      rigid_shift_thread(connect_to_rigid_shift_publisher, settings.shift_address);

   //wait until we disconnect from the systems
   linac_thread.join();
   if (settings.using_DIR)
      mr_thread.join();
   else
      rigid_shift_thread.join();
}
```

Client @MR-linac: linac thread

```
function connect_to_linac(address)
{
```

```
    previous_cp; // control point
    current_cp;

    // set routing key to segment.name_of_linac
    routing_key = "segment.U01"

    linac_handle = connect(address);

    linac_message;

    while (linac_handle.connected)
    {
        if !(linac_handle.get_message(linac_message, time_out))
            continue; // timed_out

        current_cp.parse_message(linac_message);

        if !(current_cp.MU - previous_cp.MU > 0.001)
            continue; // no MU delivered

        // shift and irradiated anatomy are updated by the other thread
        msg = create_protobuf_message(current_cp, previous_cp, shift, irradiated_anatomy);
        publish_to_rabbitMQ(msg, routing_key);

        previous_cp = current_cp;
    }
}
```

Client @MR-linac: mr thread

```
function connect_to_mr(address)
{
    slice;
    dynamic_index = 0;

    //set routing key to images.name_of_linac
    routing_key = "images.U01";

    mr_handle = connect(address);
    mr_message;

    while (mr_handle.connected)
    {
        if !(mr_handle.get_message(mr_message, time_out))
            continue; // timed_out

        slice.parse_message(mr_message);

        // check if this is a new 3D volume
        if (slice.dynamic > dynamic_index)
        {
            dynamic_index = slice.dynamic;
            update_irradiated_anatomy(dynamic_index);
        }
```

```
        // shift and irradiated anatomy are updated by the other thread
        msg = create_protobuf_message(slice);
        publish_to_rabbitMQ(msg, routing_key);
    }
}
```

Client @MR-linac: rigid shift thread

```
function connect_to_rigid_shift_publisher(address)
{
    shift = [0.0, 0.0, 0.0];

    handle = connect(address); // CMM or phantom
    message;

    while (handle.connected)
    {
        if !(handle.get_message(message, time_out))
            continue; // timed_out

        shift.parse_message(message);

        update_shift(shift);
    }
}
```

For the worker we have the following pseudocode. The displaying of the data is done using a user interface made with QT [5], the pseudocode for that is left out here for brevity.

Worker: main thread

```
function main(file)
{
    settings = parse_input(file);

    handle = connect_to_rabbitMQ(address);

    segment_thread(connect_to_segment_exchange, handle);

    if (settings.using_DIR)
        images_thread(connect_to_images_exchange, handle);

    //wait until we disconnect from the system
    live_dose_thread.join();
    images_thread.join();
}
```

Worker: segment thread

```
function connect_to_segment_exchange(handle)
{
   segment_queue;
```

```
  exchange = “segment”;

   // Spawn a new thread so that we have a dedicated thread to obtain messages
   live_dose_thread(live_dose);

   while (handle.connected)
   {
      msg = get_message_from_exchange(handle, exchange);
      if (msg.empty())
         continue;

      segment = parse_message(msg);
      push!(segment_queue, segment);
   }
}
```

Worker: live dose thread

```
function live_dose()
{
   Patient_anatomy; // tracks all dose distributions, DVHs, dynamics, and CT/density
   treatment_plan;
   structure_set;

   While (true)
   {
      // our implementation uses conditional variables and mutexes for the queue
      // this lets allows for this thread to sleep and only wake up when there is something in
      // the queue. This is simplified here with wait_until statements.
      segment = wait_until_segment(segment_queue);

      if (segment.ID != patient_anatomy.ID)
      {
         load_patient_data(segment.ID, patient_anatomy, treatment_plan, structure_set);
         setup_dose_engine(patient_anatomy, treatment_plan, structure_set);
         cumulative_MU = 0;
         planned_dose_thread(calculated_planned_dose); // calculate the intended dose
      }
      else if (segment.irradiated_anatomy != patient_anatomy.loaded_anatomy)
      {
         //warp the original CT/density to the anatomy that needs to be loaded
         //use the found DVF to warp the dose and structures
         EVolution(patient_anatomy, structure_set);

         wait_until_forecast_completed();
         setup_dose_engine(patient_anatomy, treatment_plan, structure_set);
      }

      QA_segment = get_equivalent_treatment_plan_segment(treatment_plan, segment);

      calculate_dose(patient_anatomy, segment);
      calculate_dose(patient_anatomy, QA_segment);

      forecast_thread(forecast_dose, cumulative_MU, patient_anatomy, treatment_plan,
```

```
structure_set);

        publish_or_display_results(patient_anatomy);
    }
}
```

Worker: forecast thread

```
function forecast_dose(cumulative_MU, patient_anatomy, treatment_plan, structure_set)
{
    if (already_calculating_forecast)
        return;

    remaining_treatment = get_remaining_treatment(cumulative_MU, treatment_plan);

    calculate_dose(patient_anatomy, remaining_treatment);

    update_DVHs(patient_anatomy);

    publish_or_display_results(patient_anatomy);
}
```

Worker: images thread

```
function connect_to_images_exchange(handle)
{
   exchange = "images";
   dynamics;

    while (handle.connected)
    {
        msg = get_message_from_exchange(handle, exchange);
        if (msg.empty())
            continue;

        slice = parse_message(msg);

        if (slice.patient_ID != dynamics.patient_ID)
           dynamics.reset(slice.patient_ID);

        dynamics.add_slice(slice);

        if (dynamics.completed(slice.dynamic))
        {
            add_dynamic_to_patient_anatomy(dynamics.dynamic(slice.dynamic));
        }
    }
}
```

References

[1] Denis de Senneville B, Zachiu C, Ries M, Moonen C. EVolution: an edge-based variational method for non-rigid multi-modal image registration. Phys Med Biol 2015;61;7377-96. https://doi.org/10.1088/0031-9155/61/20/7377

[2] Zachiu C, Denis de Senneville B, Tijssen R, Kotte A, Houweling A, Kerkmeijer L et al. Non-rigid CT/CBCT to CBCT registration for online external beam radiotherapy guidance. Phys Med Biol 2018;63 https://doi.org/10.1088/1361-6560/aa990e

[3] https://www.rabbitmq.com/

[4] https://protobuf.dev/

[5] https://www.qt.io/

[6] Oolbekkink S, Wolthaus J, Van Asselen B, Van den Dobbelsteen M, Raaymakers B Validation of a diode-based phantom for high temporal and spatial measurements in a 1.5 T MR-linac J Appl Clin Med Phys 2025;26. https://doi.org/10.1002/acm2.14604
